# *In Vitro* Digestibility of Minerals and B Group Vitamins from Different Brewers’ Spent Grains

**DOI:** 10.3390/nu14173512

**Published:** 2022-08-26

**Authors:** Anca Corina Fărcaș, Sonia Ancuța Socaci, Maria Simona Chiș, Javier Martínez-Monzó, Purificación García-Segovia, Anca Becze, Anamaria Iulia Török, Oana Cadar, Teodora Emilia Coldea, Marta Igual

**Affiliations:** 1 Department of Food Science, Faculty of Food Science and Technology, University of Agricultural Sciences and Veterinary Medicine of Cluj-Napoca, 400372 Cluj-Napoca, Romania; 2 Food Investigation and Innovation Group, Food Technology Department, Universitat Politècnica de València, Camino de Vera s/n, 46022 Valencia, Spain; 3 National Institute for Research and Development of Optoelectronics INOE 2000, Research Institute for Analytical Instrumentation, 67 Donath Street, 400293 Cluj-Napoca, Romania

**Keywords:** *in vitro* digestibility, bioaccessibility, brewers’ spent grain, minerals, B vitamins

## Abstract

Brewers’ spent grain (BSG), the main by-product of the brewing industry, is a rich source of minerals and water-soluble vitamins such as thiamine, pyridoxine, niacin, and cobalamin. Bioaccessibility through *in vitro* digestion is an important step toward the complete absorption of minerals and B group vitamins in the gastrointestinal system. Inductively coupled plasma optical emission spectrometry (ICP-OES) together with inductively coupled plasma quadrupole mass spectrometry (ICP-MS) was used for the quantification of the macro- and micro-minerals. An ultra-high performance liquid chromatography (UHPLC) system coupled with a diode array detector (DAD) was used for B group vitamin identification. Four different industrial BSG samples were used in the present study, with different percentages of malted cereals such as barley, wheat, and degermed corn. Calcium’s bioaccessibility was higher in the BSG4 sample composed of 50% malted barley and 50% malted wheat (16.03%), while iron presented the highest bioaccessibility value in the BSG2 sample (30.03%) composed of 65% Pale Ale malt and 35% Vienna malt. On the other hand, vitamin B1 had the highest bioaccessibility value (72.45%) in the BSG3 sample, whilst B6 registered the lowest bioaccessibility value (16.47%) in the BSG2 sample. Therefore, measuring the bioaccessibilty of bioactive BSG compounds before their further use is crucial in assessing their bioavailability.

## 1. Introduction

Food waste management represents a serious and ever-growing issue with important consequences for economic, environmental, and climatic changes. Even though progress has been made over time, there is an urgent demand for sustainability in the food processing chain [1]. To address this issue, the European Commission underlined the necessity of a Circular Economy Action Plan and mentioned that a sustainable proposal for circular economy might be the reuse of by-products as new raw materials [2].

Brewers’ spent grain (BSG) is the main by-product obtained from the industrial brewing process, and EU brewing alone generates annually a total amount of 3.4 million tons of BSG [3]. According to Andres et al. [4], the production of one hectoliter of beer generates 20 kilos of BSG, and the number of breweries has recently doubled in the EU, nowadays reaching a total number of 9500. Worldwide, the beer industry has undergone great growth, reaching total production and consumption rates of 1.95 billion hectoliters and 357 million hectoliters, respectively [4].

It is worth pointing out that BSG represents 85% of the brewing waste and is a valuable source of bioactive compounds such as minerals, vitamins, fiber (hemicellulose, lignin, cellulose), proteins, and amino acids [3,5]. BSG contains important amounts of B group vitamins such as thiamine (B_1_), riboflavin (B_2_), niacin (B_3_), pyridoxine (B_6_), pantothenic (B_5_), and folic acids, but also higher amounts of minerals such as calcium (Ca), sodium (Na), and magnesium (Mg), together with manganese (Mn), iron (Fe), potassium (K), and copper (Cu) [6]. A large body of studies has mentioned that BSG could be successfully used in the manufacturing of bakery and pastry products such as pasta, biscuits, cookies [7,8,9], bread [10], and extruded snacks [11], as well as meat products such as frankfurters [12] and burgers [13].

Even if the by-products are generally rich in bioactive compounds, their bioaccessibility is crucial to establish their nutritional value during the physiological digestion process [14]. Nowadays, more than two billion people all over the world are affected by different mineral deficiencies, with Fe and Zn deficiencies being the most prevalent for children [15]. Furthermore, Fe deficiency is considered the leading nutritional disorder worldwide according to Cian et al. [16], which affects over 30% of the world’s population, mainly children and women from developing countries [17]. On the other hand, Zn deficiency is considered a worldwide problem, with an approximate percentage of risk for the populations of underdeveloped countries of 33% [17]. The consumption of food with bioaccessible minerals is essential for the human body in order to perform vital functions [18]. Even if there are several factors that could lead to mineral deficiencies such as an inadequate dietary intake, their poor bioavailability remains the most likely cause [19]. On the other hand, water-soluble vitamins such as B group are essential in preventing diseases such as anorexia, beriberi, mental problems, cardiovascular diseases, depression, visual disorders, complication of diabetes mellitus, or even dermatological disorders [20]. The bioavailability of the vitamins could not be fully predicted, mainly because of their unknown bioaccessibility during the gastrointestinal system [20].

Furthermore, bioaccessibility and bioavailability are the main concepts that must be taken into consideration to evaluate the pros and cons associated with the intake of an element from the food matrix and its further nutritional value in the organism [21]. The bioaccessibility is defined as the amount of bioactive compounds that are released from the food matrix before their absorption from the small intestine [21]. The bioavailability is mentioned as the fraction of ingested biocomponents that are able to reach the blood flow and the different organs and tissues, resulting in their bioactivity [22].

In a recent study, Igual et al. showed that an *in vitro* digestion process based on a static *in vitro* method such as COST INFOGEST network could be successfully used in evaluating the bioaccessibility of some bioactive compounds from the vegetable matrix [23].

The four BSG types were collected from Romanian industrial breweries, from different types of beers. The beers could be classified according to Salanță et al. [24] on three main groups, as follows: ale beers (top or high fermentation), lager with low fermentation, and non-alcoholic beers. On the other hand, Yeo et al. [25] mentioned different categories of craft beers or specialty beers, which include low-calorie beer, non-alcoholic and low-alcohol beers, gluten free beers, and functional and novel-flavored beers, whilst [26] mentioned that the key difference between beers is the fermentation method, and classified beers in “brown beer” and “white beer”. White beer, which is also known as wheat beer, is defined by the World Cup Style Guidelines as a product made with at least 50% malted wheat, which is top fermented and with a typical aroma and flavor [26]. White beers produced by international breweries are gaining more and more attention, with one out of ten beers sold in Germany being a wheat beer [27]. Even if barley is the main cereal in brewing, maize and wheat have drawn mainstream attention, being used as beer adjuvants. For example, maize used at a percentage of 30% for beer manufacturing led to an 8% total reduction in production costs [28].

**Figure 1:**
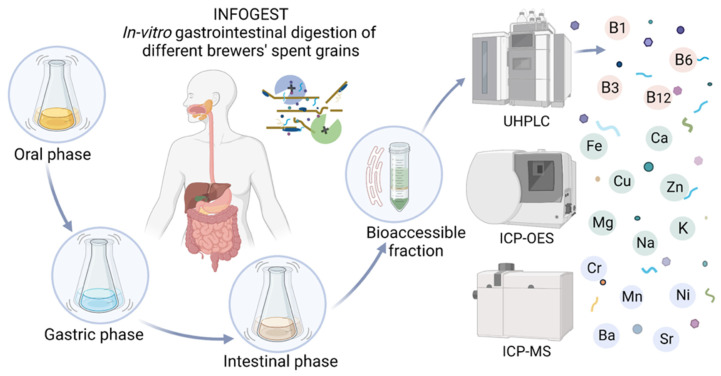
*In vitro* gastrointestinal digestion of minerals and B group vitamins from different BSG types.

Therefore, the aim of the present paper is to establish the bioaccessibility of minerals and B group vitamins of different BSG types through *in vitro* gastrointestinal digestion model. The illustrative design of the present research is displayed in Figure 1, as follows:

## 2. Materials and Methods

### 2.1. Materials

The amylase, pepsin, gastric lipase, and pancreatin were purchased from Sigma-Aldrich Inc. (Sydney, NSW, Australia). All of the used solvents were of HPLC grade and were acquired from VWR, Inc. (Radnor, Pennsylvania, PA, USA). The ultra-pure water was obtained using an ULTRACLEAR UV UF EVOQUA purification system, (Erlanger, Kentucky, AL, USA). The multi-elemental solutions of 1000 mg/L ICP Standard Certipur^®^ were purchased from Merk, (Darmstadt, Germany). All of the standards used were from Sigma-Aldrich (Sydney, NSW, Australia). 

### 2.2. Brewers’ Spent Grain from Different Types of Malt

The brewers’ spent grain samples were kindly obtained from Romanian industrial breweries, which were dried for 12 h and milled using a professional laboratory mill (IKA A10, Staufen, Germany), as previously described by our research group [3]. The codification of the 4 by-products resulting from beer processing, the raw materials used, and the roasting treatments are displayed in Table 1.

### 2.3. Simulated Gastrointestinal Digestion

The sample digestibility was assessed using the standardized static *in vitro* digestion method suitable for food (COST INFOGEST network) proposed by Minekus et al. [29] and Brodkorb et al. [30]. This *in vitro* method is a general, standardized, practical static digestion procedure based on relevant conditions that can be applied for various purposes. The aim of this procedure was to harmonize the *in vitro* static systems that simulate digestive processes by defining the key parameters and conditions.

The steps for the *in vitro* digestion protocol were: oral phase, mixing the sample and simulated salivary fluid (SSF) (1:1) with amylase (75 U/mL) at pH 7 for 2 min; gastric phase, mixing the oral bolus and simulate gastric fluid (SGF) (1:1) with pepsin (2000 U/mL) and gastric lipase (60 U/mL) at pH 3 for 2 h; intestinal phase, mixing the gastric chyme and simulate intestinal fluid (SIF) (1:1) with pancreatin (Trypsin activity 100 U/mL) at pH 7 for 2 h; filtration, centrifuging at 4500× *g* rpm for 30 min and then filtering through a 1 µm glass fiber membrane. 

The concentration of enzymes used was estimated according to the activity certified by the manufacturer’s analysis. The simulated fluids contained KCl, KH_2_PO_4_, NaHCO_3_, NaCl, MgCl_2_ (H_2_O)_6_, (NH_4_)_2_CO_3_, CaCl_2_, and HCL or NaOH for the pH adjustment of the stock solutions of simulated digestion fluids. The *in vitro* sample digestibility (IVD%) value was calculated as the difference between the initial sample and the undigested one; the obtained result was divided by the initial mass sample and multiplied by 100, as mentioned by [23,31].

The analyses were performed in triplicate. The blank and BSG samples were subjected to *in vitro* digestion, and the samples were collected according Minekus et al. [29] and Brodkorb et al. [30] and then freeze-dried using a protease inhibitor (Pefabloc SC, Sigma-Aldrich) with the ability to irreversibly inhibit trypsin and chymotrypsin. The blank and BSG digested samples were analyzed in triplicate for mineral and B group vitamins. The analyzed compounds present in the water and reagents were also analyzed and corrected in the final fraction. The bioaccessibility was calculated according to the proposed equation used by Khouzam et al. [32] as being the ratio between the concentration of bioactive compounds after *in vitro* digestion and the initial concentration of the same bioactive compounds before starting the *in vitro* digestion process.

### 2.4. Mineral Determination

In order to determine the major (Na, K, Ca, Mg, Fe, Cu, Zn) and trace element concentrations (Co, Cr, Mn, Ni, Ba, Sr, Rb), an Xpert closed-vessel system (Berghof, Eningen, Germany) was used for sample digestion. Here, 200 mg of sample was digested using 10 mL HNO_3_ 65% and 2 mL H_2_O_2_ 30% in polytetrafluoroethylene digestion vessels, using a four-step digestion program (120 °C and 170 °C—heating; 100 °C and 25 °C—cooling) for a total digestion time of 25 min. After, the vessels were cooled down and the volume was made up with ultrapure water to 20 mL. The resulting solutions were analyzed using an Optima 5300DV inductively coupled plasma optical emission spectrometer (ICP-OES, Perkin Elmer, Norwalk, CT, USA) for the determination of the major elements, and an ELAN DRC II inductively coupled plasma quadrupole mass spectrometer (ICP-MS, Perkin–Elmer, Waltham, MA, USA) for the determination of the minor elements. The calibration standards were prepared from ICP Multi-Element Standard Solution IV (1000 mg/L) (Merck, Darmstadt, Germany) for the ICP-OES and Multi-Element Calibration Standard 3 (10 mg/L) (Perkin Elmer Pure Plus, Waltham, MA, USA) at appropriate dilutions. The detection limits for the ICP-OES and ICP-MS are mentioned in the Supplementary Files, Table S1.

### 2.5. B Vitamins Analysis

For sample extraction, 0.5 g of sample was extracted with 1 mL of ultra-pure H_2_O for 20 min in an ultrasonic bath (SONOREX, Bandelin, RK 103H, Darmstadt, Germany) at room temperature. The samples were centrifuged (Microcentrifuge Hettich D-78532, Germany) at 11,000× *g* rpm for 2 min, then the supernatant was filtered through a 0.45 µm cellulose filter.

A Vanquisher H UHPLC (ultra-high performance liquid chromatography) system from Dionex (Thermo Fisher Scientific, Germany) with a DAD detector was used for the analysis of the following vitamins: thiamine (B1), riboflavin (B2), nicotinamide (B3), pyridoxine (B6), cyanocobalamin (B12). The mobile phase was composed of ultra-pure H_2_O with 1% acetic acid and MeOH in gradient with a flow of 0.3 mL/min. The chromatographic column used was a Accucore aQ (100 × 2.1 mm, 2.6 mm) from Thermo Fisher kept at 25 °C. The injection volume was 8 µL and the detector was set at 270 nm. Thiamine hydrochloride, riboflavin, niacinamide, pyridoxine hydrochloride, and cyanocobalamin standards were purchased form Sigma-Aldrich, Inc. (Sydney, NSW, Australia). The dilutions were made in ultra-pure water with a 0.1–100 µg/mL linearity range. The quantification limit was 0.1 and the detection limit was 0.03 µg/mL for all analyzed vitamins.

### 2.6. Statistical Analysis

An analysis of variance (ANOVA) with a 95% confidence level (*p* < 0.05) using the Statgraphics Centurion XVIII Software (Statgraphics Technologies, Inc., The Plains, VA, USA), version 18.1.13, was applied to evaluate the differences between the studied samples. The method used to discriminate between the means was Fisher’s least significant difference procedure. All samples were made in triplicate and each result is expressed as the mean between them followed by the standard deviation. Small letters indicate homogeneous groups established by the ANOVA (*p* < 0.05) by comparing all samples (BSGs) before and after *in vitro* digestion, whilst capital letters indicate homogeneous groups established by the ANOVA (*p* < 0.05) by comparing samples before and after the *in vitro* digestion for each BSG sample.

## 3. Results

A total number of 18 minerals were determined, from which minerals such as cobalt (Co), arsenic (As), cadmium (Cd), mercury (Hg), lead (Pb), and rubidium (Rb) were below the detection limit (0.10 mg/kg).

The identified minerals were divided in two groups, as follows: macro-minerals such as calcium (Ca), potassium (K), sodium (Na), and magnesium (Mg); micro-minerals such as iron (Fe), zinc (Zn), manganese (Mn), copper (Cu), chromium (Cr), nickel (Ni), barium (Ba), and strontium (Sr). Macro-minerals are defined as minerals required in amounts greater than 100 mg/day, while micro-minerals, also entitled trace elements, are required in amounts of less than 100 mg/day [15].

**Figure 2:**
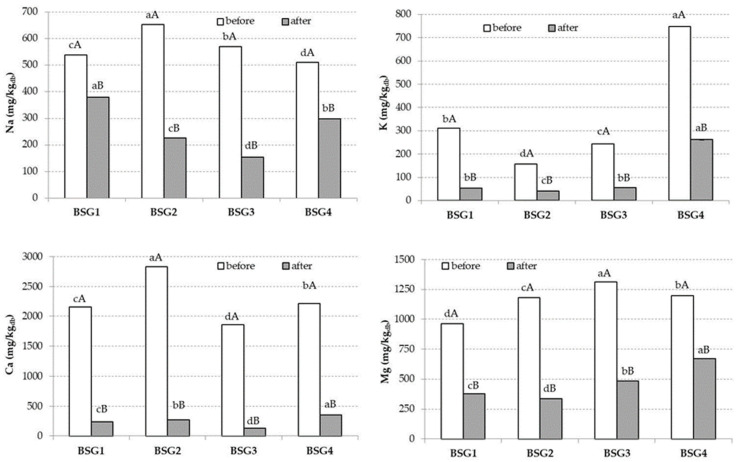
Mean values and standard deviations (error bars) of the macro-mineral (Na, K, Ca, and Mg) contents in four different brewers’ spent grain (BSG) samples. For each mineral, small letters indicate homogeneous groups established by the ANOVA (*p* < 0.05) by comparing BSGs before and after *in vitro* digestion. For each mineral, capital letters indicate homogeneous groups established by the ANOVA (*p* < 0.05) by comparing samples before and after *in vitro* digestion for each BSG sample. Note: db, dry weight.

With respect to the macro-minerals, Ca was the main mineral identified in BSG2 with a value of 2834.30 mg/kg (Figure 2), while Mg (Figure 2) was identified in bigger amounts in the BSG3 sample.

Regarding the Na and K contents in the studied BSG types, the values ranged between 509.93–652.82 mg/kg and 157.74–747.48 mg/kg, respectively. Similarly, Fărcaș et al. [3] showed that the Na and K contents could greatly vary between samples. For instance, Na range was 203.38–226.35 mg/kg and K was identified in a range of 486.14–514.37 mg/kg.

**Figure 3:**
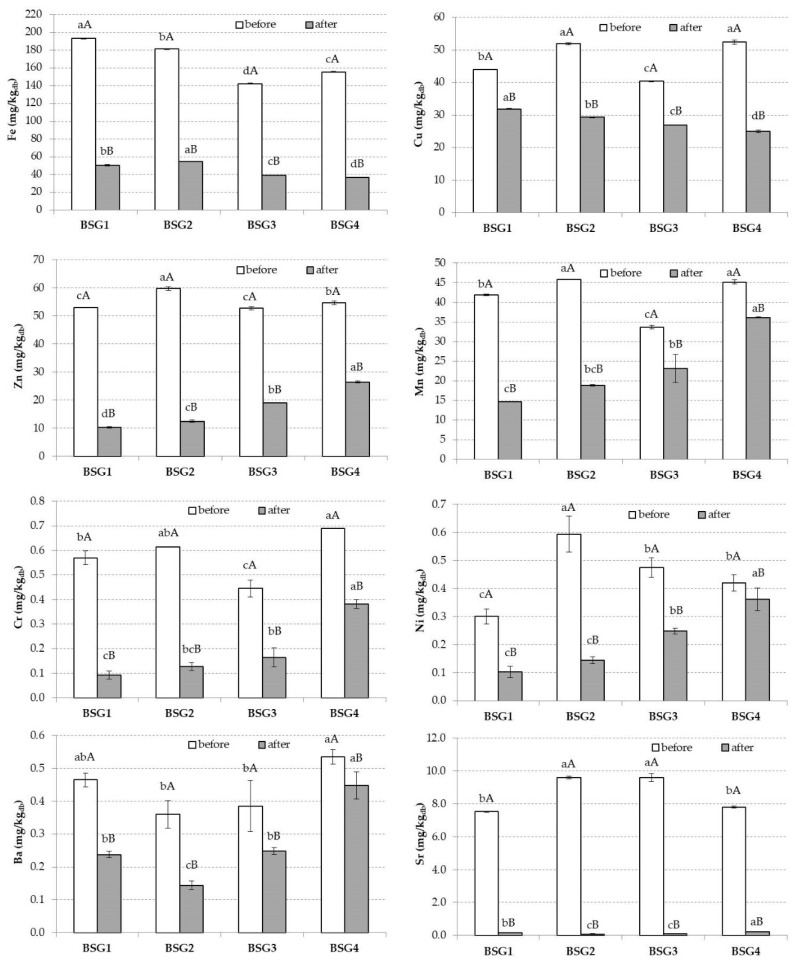
Mean values and standard deviations (error bars) of the micro-mineral (Fe, Cu, Zn, Mn, Cr, Ni, Ba, and Sr) contents in four different brewers’ spent grain (BSG) samples. For each mineral, small letters indicate homogeneous groups established by the ANOVA (*p* < 0.05) by comparing BSGs before and after *in vitro* digestion. For each mineral, capital letters indicate homogeneous groups established by the ANOVA (*p* < 0.05) by comparing samples before and after *in vitro* digestion for each BSG sample. Note: db, dry weight.

In the present study, Fe reached the highest value in the BSG1 sample (Figure 3), whilst Zn was the main representant of the BSG2 sample, with a value of 59.79 mg/kg, as illustrated in Figure 2. Mn reached higher extended values in BSG2 and BSG4 (Figure 2), whilst Cr was significantly higher in BSG4, with a value of 0.69 mg/kg (Figure 3). Sr reached similar values in BSG2 and BSG3 (Figure 3), while the Ba values were not statistically different between samples (*p* < 0.05) (Figure 3).

For BSG4, bioaccessibility of Zn, Ca, and Mg reached values of 48.35%, 16.03%, and 56.07%, respectively, while the BSG2 sample registered lower values of 20.85%, 9.53%, and 28.44%, respectively. The bioaccessibility of Na was the highest in the BSG1 sample (70.43%), while a value of 58.43% was achieved in the BSG4 sample.

On the other hand, for BSG1, Cu bioaccessibility registered a statistically significant difference (*p* < 0.05) with a value of 72.42%, while BSG4 sample reached a value of 47.80%. With respect to Cr bioaccessibility, the highest value of 55.23% was obtained in the BSG4 sample, whilst a value of 16.05% was registered for BSG1 sample. 

**Figure 4:**
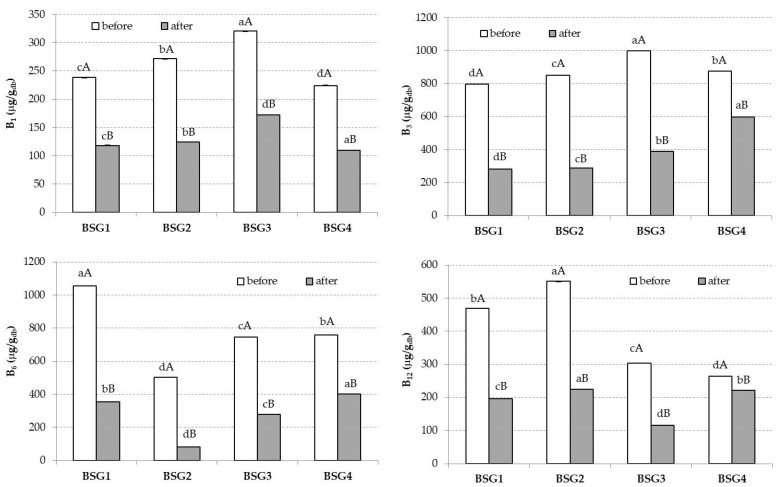
Mean values and standard deviations (error bars) of B group vitamin (B1, B3, B6, B12) contents in four different brewers’ spent grain (BSG) samples. For each mineral, small letters indicate homogeneous groups established by the ANOVA (*p* < 0.05) by comparing BSGs before and after *in vitro* digestion. For each mineral, capital letters indicate homogeneous groups established by the ANOVA (*p* < 0.05) by comparing samples before and after *in vitro* digestion for each BSG sample. Note: db, dry weight.

With respect to B group vitamins (B1, B3, B6, B12), their values before and after digestion are illustrated in Figure 4. The highest amount of vitamin B1 was reached in the BSG3 sample (Figure 4) with a value of 320.55 µg/g, whilst vitamins B6 and B12 were the highest in BSG1 and BSG2 samples, with values of 1057.36 µg/g and 550.93 µg/g, respectively (Figure 4). After the *in vitro* digestion, all analyzed vitamins decreased their values, as presented in Figure 3. Regarding their bioaccessibility, vitamin B1 had a value of 72.45% in the BSG3 sample, while a value of 68.44% was found for vitamin B3 for the BSG4 sample. Vitamin B6′s bioaccessibility ranged from 16.47 to 52.94% for the BSG2 and BSG4 samples, respectively. Vitamin B12 registered values of 41.76%, 40.81%, 38.10%, and 83.57% for the BSG1, BSG2, BSG3, and BSG4 samples, respectively. 

## 4. Discussion

Bioaccessibility is defined as the rate-limiting step that influences the overall bioavailability of numerous bioactive compounds, as well as the portion released from the matrix and the amount available for further absorption [33]. The good reproducibility, lower cost, ease of sampling, labor and time savings, and lack of ethical restrictions for bioactive compounds are some of their advantages compared to in vivo studies [33].

The rich BSG mineral content was also highlighted by Jackowski et al. [34], who mentioned that the BSG ash amounts varied over a range of 1.1–4.6%, while Chetrariu and Dabija emphasized a value range of 2% to 6% for BSG ash [35]. Bonifácio-Lopes et al. highlighted that the Ca in BSG was identified within a range of 2200–3515 mg/kg, Mg at 1900–2400 mg/kg, and Na at 258.1–700 mg/kg [36]. In the present study, BSG Ca amounts ranged between 1857.4 and 2834.5 mg/kg, Mg was identified within a range of 963.79–1311.75 mg/kg, and Na at 537.11–569.66 mg/kg. It is worth pointing out that the BSG contents for almost all the macro-minerals and micro-minerals were statistically significantly different (*p* < 0.05) between samples (Figure 2 and Figure 3). For instance, Ca, K, Na, and Mg for BSG3 of 1857.4 mg/kg, 242.64 mg/kg, 596.66 mg/kg, and 1311.75 mg/kg, respectively, while BSG4 had for the same macro-elements values of 2211.5 mg/kg, 748.48 mg/kg, 509.93 mg/kg, and 1196.08 mg/kg, respectively. Therefore, we can conclude that the tested BSG samples had variable mineral contents, leading to possible different influences on human health. The differences between the different BSG mineral contents could be explained by the processing method, or even the barley type used during malting, the harvesting and cultivation times, and the different adjuncts used during wort manufacturing [37,38].

Ca is a macromolecular nutrient that is necessary for the development, maintenance, and growth of human bone, and its deficiency leads to several diseases such as osteoporosis. Mg is an essential element that activates more than 300 enzymes involved in protein, nucleic acid, and carbohydrate metabolism, being essential for bone mineralization and development [39]. 

Fe is a micromineral that is indispensable for life, being involved in numerous physiological processes such as energy metabolism, oxygen transport, enzymatic activity, and even for the metabolic demands of renal cells [40]. Cr is considered an essential nutrient due to its presence in the active centers of several enzymes, being often used in the manufacture of different mineral or multivitamin supplements, with an RDA (recommended daily allowance) of 200 µg/day for adult women and men. Furthermore, Cr might be involved in the glucose transport from the blood to the cells, having positive effects on reducing blood sugar levels and being essential for complete glucose metabolism in humans [41,42].

On the other hand, Ni is an essential micro-nutrient for plants, having a main role in numerous biological mechanisms, and its deficiency can affect a plant’s nitrogen metabolism and Fe absorption [43]. Ni also plays a key role in some enzymes, being an essential component [44]. Recently, Rosa et al. [45] mentioned that the WHO and FAO established a permissible limit for the Ni content in edible vegetable sources of 1.63 mg/kg, but our results are below the limit (0.30–0.60 mg/kg). Sr is recommended in some countries as a supplement for enhancing bone mineral density [46], whilst Zn is an essential mineral for the development and growth of all organisms, playing a main role in different cellular functions such as cell growth and division, cellular transport, and immune and endocrine systems [47]. 

As highlighted by Bohn et al. [48], there are several factors involved in mineral availability during digestion, such as oxidation and reduction or even complexation reactions. The bioaccessibility of minerals could be affected by their different chemical forms or by the presence of antinutritional substances that might interfere with their essential mineral absorption [18]. For instance, phytic acid, also entitled *myo*-inositol-hexaphosphate, is an antinutrient that was claimed to be present in brewers’ spent grain and which is able to bind minerals and proteins, thereby influencing their solubility, digestibility, functionality, and absorption [49]. In line with this, Lynch et al. [50] mentioned that BSG has a high level of phytic acid, with a negative involvement in mineral solubility. 

Moreover, it was claimed that *myo*-inositol phosphates could form insoluble complexes with divalent cations such as Ca^2+^, Fe^2+^, Mg^2+^, Cu^2+^, Mn^2+^, and Zn^2+^, having a highly negative influence on mineral absorption [18]. Furthermore, fiber could be another factor that might possibly lead to low mineral absorption [18], with BSG containing about 35–60% fiber on a dry weight basis according to Puligundla et al. [51]. This idea is also sustained by Rousseau et al. [17], who emphasized fiber’s capacity to bind minerals, forming fiber–mineral complexes, which are impossible to hydrolyze via human digestion. Fiber’s specific characteristics, such as its solubility, might be the key factors that influence the mineral bioaccessibility level [17]. For instance, from the insoluble fiber types, lignin showed the greatest mineral-binding capacity as compared with hemicelluloses and cellulose, probably because of its polyphenolic nature [17]. In addition to fiber’s capacity to bind minerals, it seems that its negative influence is also related to its capacity to influence the direct contact between nutrients and digestive enzymes, thanks to its increased digestive viscosity [52]. It should also be mentioned that BSG contains tannins [53], which could also be involved in mineral availability [18]. According to a recent study by Lemmens et al. [54], during human gastrointestinal digestion, phytate and arabinoxylan’s cell wall materials resist, resulting in very low mineral bioaccessibility. Therefore, the initial chemical composition of the raw materials is one of the most important factors involved in their mineral bioaccessibility, mainly their antinutrients factors and their chelate digestibility (an antinutrient mineral complex) [17]. Moreover, it was highlighted that two main factors, i.e., the two different corn genotypes and the environmental conditions, could directly influence the phytate content in corn [55].

Cereal malting is also an important process, which involves germination followed by heat treatment, which generally lead to an increased nutritional content and degree of digestibility [19]. During malting, the mineral bioaccessibility could increase. For instance, the bioaccessibility of Ca and Mn in wheat (*Triticum aestivum*) increased through malting by 24% and 42%, while the bioaccessibility of Ca in barley remained the same during malting. This is in line with our result, where the biggest Ca bioaccessibility value was reached in the BSG4 sample (16.03%), composed of 50% malted barley and 50% malted wheat. With respect to Mn’s bioaccessibility, the biggest value was reached in the BSG4 sample, followed by the BSG3 sample, with values of 80.08% and 68.81%, respectively.

It is of note that the bioaccessibility of Fe and Zn significantly increase during the germination process, mainly due to the phytate breakdown. For instance, wheat germination increased Fe and Zn’s bioaccessibility from 4.6% and 2.5% to values of 14.1% and 14.6%, respectively [53]. Moreover, Lemmens et al. [54] showed that hydrothermal treatment could lead to an almost complete phytate breakdown, demonstrating that the Fe ions were no longer bound to the phytate structures after the treatment. In the present study, the highest Fe bioaccessibility was 30.03% for the BSG2 sample, whilst BSG4 presented the biggest value of 48.35% for Zn. This could also be justified by the heat treatment parameters, such as the malted kilning temperature, which could contribute to a reduction in antinutritional factors such as tannins, polyphenols, and phytic acid. Increased Fe bioaccessibility after heating was also mentioned by Raes et al. [56].

The absorption of Fe and Zn mainly happens in the small intestine, where Zn is absorbed by a carrier (ZnT-1)-mediated mechanism and non-heme Fe (identified in vegetable matrix) is transported across the mucosal membrane by divalent metal transporter 1 (DMT1), together with protons [17].

The low bioaccessibility of Ca (6.82%), Na (27.03%), and K (22.69%) for the BSG3 sample could be explained by the fact that corn was present at a percentage of 30% in the sample, and it was not previously malted. Even if corn is a rich source of minerals, it also contains some antinutritional factors such as trypsin, phytase inhibitors, and α-amylase, which might be involved in digestibility inhibition, absorption, and nutrient assimilation [28]. Additionally, for corn, there is a need for further processing in the beer industry, such as the removal of the oil-rich germ and bran, aiming to avoid poor foam formation [25].

BSG also contains water-soluble vitamins such as B1, B2, B3, B5, and B6 [52], which in the present study showed decreased values after the *in vitro* digestion. For instance, vitamin B1 in the BSG1 sample had an initial value of 238.52 µg/g d.w., and after the *in vitro* digestion its value decreased, registering a final amount of 117.85 µg/g d.w. (Figure 4). In line with this, vitamin B12 had the highest value in the BSG2 sample (550.9 µg/g d.w.), and after the *in vitro* digestion its value decreased more than twice, registering a final value of 224.83 µg/g d.w. (Figure 4). According to Yaman et al. [20], several factors could negatively affect the water-soluble B vitamins, such as the temperature, pH, dietary fiber, and grinding process, or even the polypeptides, polysaccharides, and antioxidants, but also the presence of metal ions and different digestive enzymes inhibitors.

Vitamin B1 is the most heat-responsive of all B group vitamins, and its bioaccessibility could decrease with the decreasing particle size of the dietary fibers. For instance, Kurek et al. [57] showed that vitamin B1′s bioaccessibility decreased from 69.1% to 91.2% with the decreasing dietary fiber particle size from 280 μm to 100 μm. Moreover, vitamins B3 and B6 bioaccessibility are also influenced by the particle size of the dietary fiber. In this idea, Akça et al. [58] recently showed that the bioaccessibility of vitamins B1, B2, and B3 is significantly influenced by the pH of the gastrointestinal tract, different polypeptide and polysaccharides bond, temperature, and stability. Moreover, kilning barley malt process is able to increase vitamins B1 and B2 according to Hassani et al. [59].

In the present study, the highest B1 bioaccessibility value was achieved in the BSG3 sample (72.45%), probably because wheat has high *in vitro* bioaccessibility in all parts of the seed, as previously mentioned by Zaupa et al. [60]. The authors mentioned *in vitro* bioaccessibility values for vitamin B1 ranging from 62.64% to 99.65%, with the highest value being quantified in the inner part of the aleurone wheat layer. On the other hand, the bioaccessibility of vitamin B3 in wheat ranged from 10.61% to 55.94%. In good agreement with this observation, our results showed that the bioaccessibility of vitamin B3 in the BSG3 sample was lower than for vitamin B1 (33.89%), probably because in the aleurone part, niacin forms inclusions with proteins, influencing their bioaccessibility [60].

Vitamin B6 could be present in food in several forms, including as pyridoxal, pyridoxine, pyridoxamine, or even pyridoxine-glucoside. The active form of vitamin B6 is pyridoxal 5-phosphate (PLP), which is considered a coenzyme for more than 100 metabolism and biochemistry enzymes [61]. Vitamin B6 is strictly correlated with proteins, having a non-covalently bond, and its separation is related to the pH values from the gastric and intestinal phases. Therefore, it can be stated that the bioaccessibility of PLP in adults is not as affected as that in infants, considering that adults have a lower pH value [61]. Our results showed that the biggest bioaccessibility value for vitamin B6 was 52.94% for the BSG4 sample, probably because wheat sprouts have greater amounts of B group vitamins than barley sprouts. Moreover, wheat sprouts contain pantothenic acid, which was not identified in barley sprouts [62].

Vitamin B12 is an essential water-soluble vitamin, being more resistant to heat treatment compared with other vitamins of the B group [20]. It has a leading role in cellular metabolism, acting as cofactor in several metabolic processes such as mitochondrial metabolism and DNA methylation and synthesis. Several diseases such as megaloblastic anemia or even neuropsychiatric ones could derive from vitamin B12 deficiency [63]. In the oral phase, vitamin B12 starts to be bound to haptocorrin, a specific protein from saliva and gastric juice, but in duodenum, due to the presence of pancreatic proteases, the complex is disrupted [64]. It could also be released in the gastric phase by hydrochloric acid and pepsin [20]. In the present study, the bioaccessibility of vitamin B12 resulted in similar values for BSG1 (41.79%) and BSG2 (40.81%), whilst BSG4 and BSG3 reached values of 83.57% and 38.10%, respectively. The lowest value was found for BSG3, which could be explained perhaps by the fact that the corn used in the beer manufacturing was not previously malted. Moreover, the amount of dietary fiber could be involved in decreasing the B group vitamins’ bioaccessibility, since the hydroxyl group could react inside dietary fiber with the B group vitamins [65].

Therefore, the bioaccessibility of bioactive compounds such as minerals and vitamins is mainly related to the chemical composition of the food matrix. In this direction, several advances have been made over time, trying to increase the bioaccessibility and bioavailability of several food products before their in vivo or *in vitro* digestion. For instance, the use of solid-state fermentation in cereal or pulse by-products highly increased the phenolic bioaccessibility and bioavailability [66], whilst the germination increased the bioaccessibility of Ca, Fe, and Zn in pearl millet [67]. Additionally, Rousseau et al. [17] mentioned that milling, fermentation, dehulling, soaking, and thermal processing are other technological processes that could enhance the mineral bioaccessibility and bioavailability of plant-based foods, and Liao et al. [68] (Liao et al. 2022) showed that vitamin encapsulation might lead to a bioaccessibility improvement.

## 5. Conclusions

This study revealed that the contents of minerals and B group vitamins are affected by *in vitro* digestion. Micro-minerals such as Fe, Cu, Zn, Cr, Mn, Ni, Ba, and Sr and macro-minerals such as Ca, Mg, Na, K, and B group vitamins (B1, B3, B6, B12) presented different bioaccessibility values. From the macro-minerals, Na presented the biggest value (70.43%) for BSG1, whilst Cr presented the lowest bioaccessibility value (16.05%) for the same sample. Vitamin B12 showed the highest bioaccessibility (83.57%) in the BSG4 sample, followed by vitamin B1 with 72.45% bioaccessibility for the BSG3 sample and vitamin B3 with 68.44% bioaccessibility for the BSG3 sample and 52.94% bioaccessibility for the BSG4 sample.

To conclude, we can state that B12 and B1 have higher bioaccessibility levels compared with vitamins B3 and B6. The different BSG types and their chemical compositions (protein, fiber), together with the gastrointestinal pH system, temperature, and bound polypeptides and polysaccharides, are the main factors involved in the bioaccessibility of minerals and B vitamins.

Measuring bioactive compounds’ bioaccessibility is a key factor in the manufacture of fortified foods containing different bioactive compounds, such as minerals and vitamins. New strategies need to be identified to increase the bioaccessibility of the bioactive compounds in brewers’ spent grain before *in vitro* digestion. For instance, the use of modern extraction methods or the treatment of BSG with different enzymes before the digestion process might lead to a better improvement of the bioactive compounds’ bioaccessibility. This research will shed light for further studies regarding the bioaccessibility of the minerals and B group vitamins in brewers’ spent grains, with applications in several industries such as in food, pharmacology, or even cosmetics.

## Figures and Tables

**Table 1:** Types and codification of the selected brewers’ spent grain samples.

Cereal By-Products	Used Grains	Percent	Kilning Temperature °C
BSG 1	Pale Ale Malt	40%	80–85
	Munich Malt	30%	100–105
	Caramel Malt	24%	220
	Black Malt	6%	235–250
BSG 2	Pale Ale Malt	65%	80–85
	Vienna Malt	35%	100–110
BSG 3	Pilsner Malt	70%	80–85
	Degermed Corn	30%	-
BSG 4	Pilsner Malt	50%	80–85
	Wheat Malt	50%	72–80

## Data Availability

Not applicable.

## Supplementary Materials

The following supporting information can be downloaded at: https://www.mdpi.com/article/10.3390/nu14173512/s1, Table S1: Limits of detection for mineral determination by ICP–OES and ICP-MS.
