# Supplementary material for: In Vitro Digestibility of Minerals and B Group Vitamins from Different Brewers’ Spent Grains
Source: Nutrients. 2022 Aug 26;14(17):3512. doi: 10.3390/nu14173512 (PMC9460495; doi:10.3390/nu14173512)
Supplement: Supplementary file 1 [file nutrients-14-03512-s001.zip › nutrients-1878454-supplementary.pdf]

Table S1. Limits of detection for mineral determination by ICP–OES and ICP-MS

**Element**	**LOD[1] (ICP-OES) (mg kg^{-1}) [1]**	**Element**	**LOD[1] (ICP-MS) (mg kg^{-1}) [1]**
Na	0.80	Co	0.015
K	0.30	Cr	0.022
Ca	0.40	Ni	0.013
Mg	0.90	Ba	0.022
Fe	0.80	Sr	0.013
Cu	0.40	Rb	0.021
Zn	0.50	As	0.027
Mn	0.50	Cd	0.007
		Hg	0.013
		Pb	0.011

[1] Limits of detection in solid calculated for 200 mg digested sample made up to 20 mL. ICP-OES: Inductively Coupled Plasma Optical Emission Spectrometer; ICP-MS: Inductively Coupled Plasma Quadrupole Mass Spectrometer
